# Supplementary material for: Reading the family: A constructivist grounded theory on organ donation conversations
Source: PLoS One. 2024 Dec 23;19(12):e0312462. doi: 10.1371/journal.pone.0312462 (PMC11666057; doi:10.1371/journal.pone.0312462)
Supplement: S1 Appendix — (DOCX) [file pone.0312462.s002.docx]

**S2 Appendix Data collection tools**

***Observations***

| Observation questions | |
| --- | --- |
| How is approaching the families for organ donation experienced? | C1 and C2 |
| Who are the professionals involved in the process and how? | C1 and C2 |
|  |  |

***Interviews/focus groups with healthcare professionals n=71***

| Initial organ donor coordinators questions | |
| --- | --- |
| How do you experience the encounters with the families? | C1 and C2 |
| What is your role in these approaches? | C1 and C2 |
| What do you think are the most difficult aspects of these conversations? | C1 and C2 |
|  |  |

| Refining Reading the family concept: organ donor coordinators questions  (*) Questions generated from the initial/focused coding | |
| --- | --- |
| What is the reason for the difference in timing approaching? What is your motivation behind these two ways? | C1 and C2 |
| Does the approaching timing obey to what goals: support families or obtain donors? How do you deal with it | C1 and C2 |
| Do you feel that you read families’ emotions? If so, how? | C1 and C2 |
| How do you perceive organ donation conversations? Do you perceive it as a negotiation? How? Why? What does it mean? | C1 and C2 |

| Refining Reading the family concept: healthcare professional questions  (*) Questions generated from the initial/focused coding | |
| --- | --- |
| How do you perceive your role within the organ donation process? | C1 and C2 |
| How do you feel the organ donation process is carried out within the hospital? | C1 and C2 |
| How do you perceive the role of coordinators when approaching families? | C1 and C2 |
| How do you perceive organ donation conversations? Do you perceive it as a negotiation? How? Why? What does it mean? | C1 and C2 |

***Composition of focus groups (FG) with healthcare professionals n=11***

| Focus Group | N | Setting |
| --- | --- | --- |
| FG1 | 4  A&E staff | C1 |
| FG2 | 2  Organ donation staff | National level |
| FG3 | 4  ICU staff | C1 |
| FG4 | 5  ICU staff | C1 |
| FG5 | 6  ICU staff | C2 |
| FG6 | 3  Organ donor coordinators | C1 |
| FG7 | 2  ICU staff | C2 |
| FG8 | 3  Organ donor coordinators | C2 |
| FG9 | 4  A&E staff | C2 |
| FG10 | 4  A&E staff | C2 |
| FG11 | 3  ICU | C2 |

A&E = Accident and Emergency department

ICU = Intensive Care Unit

***Interviews/focus groups with families n=20 (14 families)***

| Initial family's interviews questions | |
| --- | --- |
| How did you experience the approach to discuss organ donation? | Hospital room/Participant’s homes+ |
| How did you experience the approach as a family? What do you mean? Could you expand that, please? |  |

| Refining Reading the family concept: family questions  (*) Questions generated from the initial/focused coding | |
| --- | --- |
| Did you feel that the communication about the prognosis and impeding death was clear? How? | Participants’ homes  Three families comprising of nine* participants agreed on focus groups in their houses.  (*) One participant consented to an individual interview and also a focus group with their family unit. |
| How did you feel about the process of organ donation conversation? How did you feel about the power balance in these conversations? |  |
| How did the postmortem examinations influence your decision? Could you expand on this? |  |
| How were coordinators’ approach and support? What were the most important aspects for you? |  |

+ Two participants preferred the interviews at the hospital, while another participant suggested a quiet coffee place for the interview. The other nine preferred their houses.

***Composition of focus groups with family members (FGF) n=3***

| Focus Group | N | Setting |
| --- | --- | --- |
| FGF1 | 4 | C1 |
| FGF2 | 2 | C1 |
| FGF3 | 3 | C2 |

**INTERVIEW GUIDE**

- *Welcome:*

Thanks for accepting participating

- *Informed consent process-* queries, questions. Signed ICs
- *Start audio-recording*
- *Discussion - questions*
- *Wrapping up session*
- *Final comments*
- *End of interview/ acknowledgements/stop recording*
- *Good bye*

**FOCUS GROUP GUIDE[1]**

- *Welcome:*

Thanks for coming and participating

Explain focus group’s aim

Explain layout of session

- *Coffee break*
- *Informed consent process-* queries, questions. Signed ICs
- *Intro focus group’s dynamics*

- Agreements of participation: respect among attendees, confidentiality, disclosure. Information shared in the session will be always confidential

- *Discussion and start audio-recording - questions*
- *Wrapping up session*
- *Final comments*
- *End of focus group/ stop audio-recording/ acknowledgements*
- *Coffee*

[1] Haddow G. Focus group research. In: PGSP11016 Course Research skills in the Social Sciences. Edinburgh: PowerPoint presentation accessed through LEARN. The University of Edinburgh; 2016. 6
