# Supplementary material for: Reading the family: A constructivist grounded theory on organ donation conversations
Source: PLoS One. 2024 Dec 23;19(12):e0312462. doi: 10.1371/journal.pone.0312462 (PMC11666057; doi:10.1371/journal.pone.0312462)
Supplement: Attachment — Submitted filename: ReplytoEditor.docx [file pone.0312462.s003.docx]

Dear Dr Molina,

Thank you very much for your response and comments for my Manuscript PONE-D-24-14795 entitled " Reading the family: a constructivist grounded theory on organ donation conversations "

My gratitude to reviewers for their valuable comments, please see my reply below in italics and also added in red font within the manuscript for clarity.

When submitting your revision, we need you to address these additional requirements.

1. Please ensure that your manuscript meets PLOS ONE's style requirements, including those for file naming. The PLOS ONE style templates can be found at

*Addressed.*

2. Thank you for stating the following financial disclosure:

[The research was funded by the Agencia Nacional de Investigacion y Desarrollo, Becas Chile - PhD scholarship, Chile (Folio N. 72160141).].

Please state what role the funders took in the study. If the funders had no role, please state: ""The funders had no role in study design, data collection and analysis, decision to publish, or preparation of the manuscript.""

If this statement is not correct you must amend it as needed.

Please include this amended Role of Funder statement in your cover letter; we will change the online submission form on your behalf.

*Thank you. The financial disclosure was added to the cover letter, including, ‘’The funders had no role in study design, data collection and analysis, decision to publish, or preparation of the manuscript’’.*

3. We noted in your submission details that a portion of your manuscript may have been presented or published elsewhere. [Yes, table 1 and 2 have been already published as part of the data set of the study and acknowledged within the paper.] Please clarify whether this publication was peer-reviewed and formally published. If this work was previously peer-reviewed and published, in the cover letter please provide the reason that this work does not constitute dual publication and should be included in the current manuscript.

*Thank you for this comment. Yes, both publications were peer-reviewed and formally published in the Journal of Clinical Nursing, and I have added the following to the cover letter.*

*‘’I consider important to add these tables to illustrate participants’ demographics and dataset of the study. This does not constitute duplication due to the current manuscript reports the core process, using data not yet published and essential to conceptualise, Reading the family which explains the process of approaching families for organ donation’’.*

4. We note that you have indicated that there are restrictions to data sharing for this study. PLOS only allows data to be available upon request if there are legal or ethical restrictions on sharing data publicly. For more information on unacceptable data access restrictions, please see http://journals.plos.org/plosone/s/data-availability#loc-unacceptable-data-access-restrictions.

Before we proceed with your manuscript, please address the following prompts:

a) If there are ethical or legal restrictions on sharing a de-identified data set, please explain them in detail (e.g., data contain potentially identifying or sensitive patient information, data are owned by a third-party organization, etc.) and who has imposed them (e.g., a Research Ethics Committee or Institutional Review Board, etc.). Please also provide contact information for a data access committee, ethics committee, or other institutional body to which data requests may be sent.

b) If there are no restrictions, please upload the minimal anonymized data set necessary to replicate your study findings to a stable, public repository and provide us with the relevant URLs, DOIs, or accession numbers. For a list of recommended repositories, please see

https://journals.plos.org/plosone/s/recommended-repositories. You also have the option of uploading the data as Supporting Information files, but we would recommend depositing data directly to a data repository if possible.

We will update your Data Availability statement on your behalf to reflect the information you provide.

*Thank you for the opportunity to clarify this point. Indeed, the dataset will be available upon request because it contains potentially identifying or sensitive patient/institutional information. This implied that due to the characteristic of the sample, participant could be easily identifiable. As a result, data were managed as strictly confidential but stored in the platform DataVault of my institution, the University of Edinburgh, which contains a DOI and now provided. Apologies for the confusion before. DOI:* [https://doi.org/10.7488/8f7652c6-5699-423d-894f-3241fb863029](https://eur02.safelinks.protection.outlook.com/?url=https%3A%2F%2Fdoi.org%2F10.7488%2F8f7652c6-5699-423d-894f-3241fb863029&data=05%7C02%7C%7C2d20c7f624494fdcdfa808dcad5ef8a7%7C2e9f06b016694589878910a06934dc61%7C0%7C0%7C638575870611335943%7CUnknown%7CTWFpbGZsb3d8eyJWIjoiMC4wLjAwMDAiLCJQIjoiV2luMzIiLCJBTiI6Ik1haWwiLCJXVCI6Mn0%3D%7C0%7C%7C%7C&sdata=43MaSy%2FVeXrOSU8EOVCom0gLJus9786v2leYYZgbsx0%3D&reserved=0)

*The dataset is securely kept in Data Vault and ca be requested following University of Edinburgh Data Sharing Agreement. The Research Ethics Committee at the University of Edinburgh reviewed and approved the study, which can be also contacted for further information at hiss.ethics@ed.ac.uk*

5. Please include your full ethics statement in the ‘Methods’ section of your manuscript file. In your statement, please include the full name of the IRB or ethics committee who approved or waived your study, as well as whether or not you obtained informed written or verbal consent. If consent was waived for your study, please include this information in your statement as well.

*Thank you. Full ethics statement and consent provided by all participants were added in the methods, ethical considerations section as follows, though this was already stated in participants recruitment section*

*‘’All participants provided verbal and written informed consents before data collection began’’.*

6. Please include captions for your Supporting Information files at the end of your manuscript, and update any in-text citations to match accordingly. Please see our Supporting Information guidelines for more information: http://journals.plos.org/plosone/s/supporting-information.

*Thank you. Included at the end of the manuscript using captions as in text.*

7. Please review your reference list to ensure that it is complete and correct. If you have cited papers that have been retracted, please include the rationale for doing so in the manuscript text, or remove these references and replace them with relevant current references. Any changes to the reference list should be mentioned in the rebuttal letter that accompanies your revised manuscript. If you need to cite a retracted article, indicate the article’s retracted status in the References list and also include a citation and full reference for the retraction notice.

*Revised. To my knowledge, no cited papers have been retracted.*

[Note: HTML markup is below. Please do not edit.]

Reviewers' comments:

Reviewer's Responses to Questions

Comments to the Author

1. Is the manuscript technically sound, and do the data support the conclusions?

The manuscript must describe a technically sound piece of scientific research with data that supports the conclusions. Experiments must have been conducted rigorously, with appropriate controls, replication, and sample sizes. The conclusions must be drawn appropriately based on the data presented.

Reviewer #1: Yes

Reviewer #2: Yes

2. Has the statistical analysis been performed appropriately and rigorously?

Reviewer #1: N/A

Reviewer #2: N/A

3. Have the authors made all data underlying the findings in their manuscript fully available?

The PLOS Data policy requires authors to make all data underlying the findings described in their manuscript fully available without restriction, with rare exception (please refer to the Data Availability Statement in the manuscript PDF file). The data should be provided as part of the manuscript or its supporting information, or deposited to a public repository. For example, in addition to summary statistics, the data points behind means, medians and variance measures should be available. If there are restrictions on publicly sharing data—e.g. participant privacy or use of data from a third party—those must be specified.

Reviewer #1: No

Reviewer #2: Yes

4. Is the manuscript presented in an intelligible fashion and written in standard English?

PLOS ONE does not copyedit accepted manuscripts, so the language in submitted articles must be clear, correct, and unambiguous. Any typographical or grammatical errors should be corrected at revision, so please note any specific errors here.

Reviewer #1: Yes

Reviewer #2: Yes

5. Review Comments to the Author

Please use the space provided to explain your answers to the questions above. You may also include additional comments for the author, including concerns about dual publication, research ethics, or publication ethics. (Please upload your review as an attachment if it exceeds 20,000 characters)

Reviewer #1: Dear author,

you have conducted a very comprehensive empirical study that provides a detailed insight into the constructivist approach of: Reading the family. In my view, your empirical study is very well presented. Your qualitative empirical results are clearly to understand. There is a good balance between analytical results and empirical anchor examples.

I have some suggestions for the presentation and better comprehensibility of the empirical data.

1. Provide more detailed information about your interviews and focus groups. Create a table with the composition of the individual focus groups to make it easier to understand the composition. It would also be good to attach both the discussion guide for the focus groups and the interview guide for the interviews as supplementary material.

*Thank you. This is a very helpful comment. I have added a table into S2 Appendix that shows the data collection tools, including the composition of focus groups. Interview and focus groups guides are already included in S2.*

2. Explain in more detail how the analysis process was carried out, e.g. with a category table, which could be added as an attachment.

*Thank you for this comment. I have added Table 3 to illustrate the analysis process and how the core was developed.*

2. In the introduction, you talk about different cultures in which the topic of the organ donation process has already been investigated. In my view, you are referring more to nations (and there organ transplantation system). Culture as a term seems misleading to me, as it also refers to different organ donation practices. What seems more important to me, as you also mention in your work, are different family cultures. For example, how families see themselves as a group and how this determines the way they deal with problems. In my opinion, it would be useful to differentiate this point even more in the introduction.

*Thank you for this insight. I added two sentences highlighting this aspect if that’s ok.*

*How families experienced the organ donation process in the study was already published and hence beyond the scope of this manuscript. Please see Avilés L, Kean S, Tocher J. Ambiguous loss in organ donor families: A constructivist grounded theory. J Clin Nurs. 2023;32:6504–18.*

Kind regards

Reviewer #2: The aim of this article is to find out what the encounter between professionals and families is like at the time of requesting a donation. It develops a constructivist grounded theory based on field material collected, as well as interviews and focus groups with different agents involved in the process.

General comment: Thank you for the work, it seems to me a very interesting and relevant topic, I have learned a lot reading this work. I would like to make some considerations (some questions and ask for some clarifications):

Regarding the introduction

Contextual information on the specific training that nurse coordinators have in Chile, do they receive courses on communication skills? What are these courses like? In Spain, for example, the "Alicante Model" is used, which has also been exported to other countries (Gómez, P., & de Santiago, C. (2008). The family interview. Technique and results. In El modelo español de Coordinación y Trasplantes Aula Médica). Are there data on refusal rates?

*Thank you for your suggestions. I added a couple of sentences regarding training in Chile and recent statistics included the lack of updated data on refusal rates. I hope this provides better background of nurses’ practices and Chile.*

Regarding the methodology

I suppose the extension is a limit, but I would like you to further develop some points of the methodology that I consider very interesting. The methodology is very original, in other contexts participant observation has been proposed as a useful methodology to investigate the phenomenon of donation but very difficult to carry out due to the ethical challenges it poses (López, M. V. M. M., Nieto, E. M. M. M., & Piqueras, M. C. (2022). Permission to investigate? Reflections on the ethical requirements of participant observation in the context of the family organ procurement interview. RECERCA. Revista de Pensament i Anàlisi, 27(2), https://doi.org/10.6035/recerca.6148).

Could you please clarify the limitations, difficulties you had in carrying it out and the procedure followed?

*I added a l couple of sentences on this in the data collection section. Thank you.*

Was the analysis in C2 done at a later stage? Clarify whether data collection was done in phases or simultaneously. A graph would be nice to know the sequence of data collection. Table 2 pertains to the socio-demographic profile of the participants but I would like to know who was interviewed in depth, or who participated in the focus groups. Were the focus groups mixed between professionals and families?

*Thank you for this valuable comment. I added few sentences in the data collection section, and a table of FG composition, which may give a clearer sense of the process in S2 Appendix.*

With regard to the results

As the authors say, reading emotions is related to the cultural context, decision making is a family issue. The theory has two dimensions, indirect and direct, which refers to when and how nurses read the emotions of families for and support the decision making process of bereaved families. Is it a capacity or a skill? Is it trained with training? Exclusive to nursing? What makes nursing special to this theory? Could the ability to "read families" be extended to other professionals or is it specific to nursing competences? Do you think it is relevant for a feminised profession like nursing to take on this role? Were the coordinators mostly women? reflection on gender is needed. Finally, do you think that profession, gender, training and years of experience play a relevant role?

*Thank you for this insightful comment. A defined Reading the family as a process, and a skill that can potentially be trained. Reading the family was developed to explain how nurses interact and develop approaching families in Chile. Yet the elements of the theory, could be extended to other* *professionals if they are able to first manage their emotions on the edge in difficult conversations/interactions, which I developed as edgework emotion management. I think it is relevant for nurses as they are part of these teams worldwide and potentially beyond, absolutely.*

*I added a line regarding gender, as the sample of coordinators, although majority were women, both teams included male nurses as well, and several quotations displaying the same process, emotional awareness, emotional management and skill come from male coordinators. However, to ensure anonymisation due to small number of them, this was not gendered identified. Same in terms of experience, the group was mixed, between very experienced and novice nurses. I added a sentence at the beginning of the results section addressing, which will help to clarify this point.*

*Finally, this study was a qualitative study and hence I did not explore your suggested associations/factors. However, very experienced senior medics participated in the study and not all of them seemed to master or being aware of the relevance of emotions in their interactions. Anecdotally, relatively young but very proactive medics, who valued the so called ‘soft skills’ reported to identify, acknowledge and work with their emotions when interacting with families. Indeed, fascinating aspects that may require further investigations and maybe even a secondary analysis of existing data. I also added a sentence in the conclusion section regarding its potential applicability for other healthcare professionals.Thank you so much for pointing this out.*

There is a profound debate about whether it is ethically acceptable for the same people who have been caring for the patient to be the transplant coordinators and also make the request to the family (as is the case in Spain), or whether it is preferable for it to be an external figure (as is the case in the UK or Chile). I wonder if the reading of emotions and the family (this theory) would vary if it were not an external figure but the person who has been caring for the patient and family in the ICU. Would the reading of emotions be more profound when one has participated in the process of care in the ICU for both the patient and the family or is it necessary for this reading the distance provided by being an external nurse coordinator?

*Indeed, I’m aware of this debate. In this study I got consistent data of the development of compressed intimacy to accurately support and read families’ emotions in difficult conversations/situations, which could be further developed if staff and families share more time together. Based on this, it could potentially be used when bedside teams run the process all along without the external figures. However, the ethical implications of how staff manage families’ trust and relationship in a way that families are respected and hence alike this research, further explorations using multi-methods is needed to elucidate these boundaries and ethical practices.*

I think we could go a little deeper into trust, that "click" and that reading of the family cannot be established if there is no clinical relationship built on personal trust. I think it would be good to reflect on this, in case it is useful: Martínez-López, M. V., McLaughlin, L., Molina-Pérez, A., Pabisiak, K., Primc, N., Randhawa, G., Rodríguez-Arias, D., Suárez, J., Wöhlke, S., & Delgado, J. (2023). Mapping trust relationships in organ donation and transplantation: A conceptual model. BMC Medical Ethics, 24(1), 93. https://doi.org/10.1186/s12910-023-00965-2

*Thank you for your suggestions. Indeed, the click could be interpreted using different angles, but due to word restrictions and focus of the paper, I have not expanded this point if that’s ok.*

Congratulations because the verbatims are very well chosen, they have the ability to portray the heartbreaking moment of loss.

The importance of accompaniment for the relatives is pointed out, I think it would also be good to point out the importance of rituals (based on the values and beliefs of the patient and families) and of the farewell, as well as the work of the professionals in facilitating it.

*Thank you. This is a great point but beyond the scope of this manuscript, I’m afraid, and hence I have not expanded this point to balance other suggestions and additions to the manuscript. I hope that’s ok.*

*Nonetheless, I developed this further in the paper that conceptualised in-depth families’ experiences, also cited. Please see Avilés L, Kean S, Tocher J. Ambiguous loss in organ donor families: A constructivist grounded theory. J Clin Nurs. 2023;32:6504–18.*

I think there is a typo in the supplementary material: "The other nice preferred their houses" (appendix 2 bottom of page 2). I think they mean "the other nine".

*Thank you. I corrected the typo. Indeed, the other ‘nine’.*

Ethical issues

The presence of a researcher affects the context (given that she is an external agent who is observing). How was this issue managed? Was it taken into consideration that the presence of the researcher could lead to a refusal of the application for donation? What protective measures were taken with the participants? Was time allowed for the interview in order to allow them time to grieve? Was care for the participants taken into account because they were in a situation of special vulnerability? I would like to congratulate the reflection on the researcher's own care, which is usually not taken into account and is of utmost importance.

*Thank you. Indeed, all these aspects were considered. However, due to the word limitations, I could not expand this much. First, it was common practice from the organ donation teams to invite staff to be part of the approaching process, as part of staff education and socialisation of their role, and hence my presence only observing did not disrupt coordinators’ usual practice. Nurse coordinator led fully the conversations, and I did not intervene at any point more than with my presence. My observations focus was on nurse coordinators rather than families and hence I was introduced as I was shadowing them. In other words, families were consulted if this was ok. I also asked for verbal informed consent regularly before every observation to nurse coordinators, to make sure that in their view my presence would not affect their interactions and conversations. Nonetheless, if my presence impacted the outcome of these conversations, it is very difficult to determine and to some extend acknowledge as possible. Yet it was not the view of coordinators and hence part of the nature of the enquiry.*

*Yes. Based on data and observations, families were supported all across and their grieving process and emotional status defined all interactions, including time alone, several conversations interactions, more families’ members to be involved, etc, etc. I added few sentences in the data collection section to make these points clearer, which expanded similar comments above. Also, a line in the ethics section. I hope that’s ok and addressed your valuable comments. Thank you so much for the opportunity to expand this slightly.*

Many thanks for your careful revision and for leading this process. I hope these improvements help paper’s publication.

I am happy to answer any further comments.

Kind regards,

Dr Lissette Aviles
